# The undiscovered natural product potential of *Actinomycetes*

**DOI:** 10.1038/s41429-025-00876-x

**Published:** 2025-12-02

**Authors:** Andrés M. Caraballo-Rodríguez, Andrés Cumsille, Sarolt Magyari, Maria Taboada-Alquerque, Bahar Behsaz, Tiago F. Leão, Kirk Broders, Yasin El Abiead, Jason A. Clement, Vincent Charron-Lamoureux, Simone Zuffa, Louis-Félix Nothias, Mengzhou Hu, Christopher Leone, Sarvar A. Kakhkhorov, Beatriz Cámara, Hosein Mohimani, Pieter C. Dorrestein

**Affiliations:** 1 Skaggs School of Pharmacy and Pharmaceutical Sciences, University of California San Diego, San Diego, CA USA; 2 Collaborative Mass Spectrometry Innovation Center, Skaggs School of Pharmacy and Pharmaceutical Sciences, University of California San Diego, San Diego, CA USA; 3 Departamento de Química y Centro de Biotecnología Daniel Alkalay Lowitt, Laboratorio de Microbiología Molecular y Biotecnología Ambiental, Universidad Técnica Federico Santa María, Valparaíso, Chile; 4 Department of Plant Pathology and Wisconsin Institute for Discovery, University of Wisconsin-Madison, Madison, WI USA; 5 Institute of Microbiology, Eidgenössische Technische Hochschule (ETH) Zürich, Zürich, Switzerland; 6 School of Pharmaceutical Sciences, University of Cartagena, Cartagena, Colombia; 7 Carnegie Melon University & Chemia Biosciences Inc, Pittsburgh, PA USA; 8 Núcleo de Bioensaios, Biossíntese e Ecofisiologia de Produtos Naturais (NuBBE), Institute of Chemistry, São Paulo State University (UNESP), Araraquara, São Paulo Brazil; 9 USDA, Agricultural Research Service, National Center for Agricultural Utilization Research, Mycotoxin Prevention and Applied Microbiology Research Unit, Peoria, IL USA; 10 Baruch S. Blumberg Institute, Doylestown, PA USA; 11 Institut de Chimie de Nice, Université Côte d’Azur CNRS, Nice, France; 12 Interdisciplinary Institute for Artificial Intelligence (3iA), Côte d’Azur, Sophia-Antipolis, France; 13 Laboratory of Physical and Chemical Methods of Research, Center for Advanced Technologies, Tashkent, Uzbekistan; 14 Carnegie Melon University, Pittsburgh, PA USA; 15 Department of Computational Medicine, University of California Los Angeles, Los Angeles, CA USA; 16 Department of Pharmacology, University of California San Diego, San Diego, CA USA; 17 Center for Microbiome Innovation, University of California San Diego, San Diego, CA USA

**Keywords:** Mass spectrometry, Metabolomics

## Abstract

*Actinomycetes* have been a cornerstone species for the discovery of bioactive natural products with applications in pharmacotherapy and biotechnology. To expand the experimental evidence of their biosynthetic potential, we collected liquid-chromatography mass spectrometry untargeted metabolomics data on 948 microbial strains, mostly from *Actinomycetes*. This resulted in nearly two million MS/MS spectra, with an annotation rate of 13.3% corresponding to 2352 annotated molecules. Despite the efforts to link biosynthetic gene clusters to known molecules, most remain uncharacterized. This highlights the need for metabolomic data to bridge the gap between genomic potential and metabolite production. Although many unannotated spectra might correspond to different ion forms of the same molecule, the large amount of unknown molecules present in these datasets indicates that a significant number of natural products remain to be discovered, even within one of the most thoroughly studied sets of organisms. We provide a large metabolomics dataset as a public resource for data mining of microbial molecules and highlight its value by demonstrating the detection of edapochelins, recently discovered non-ribosomal peptides.

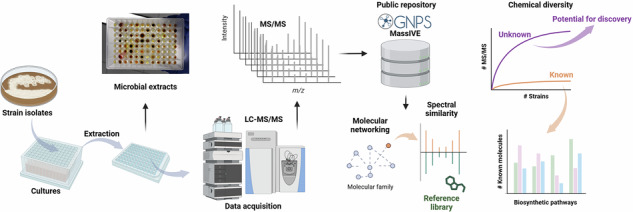

## Introduction

*Actinomycetes*, in particular the *Streptomyces* genus, continuously proves to be prolific producers of unique bioactive molecules [1–3]. The biosynthetic potential of the *Streptomyces* genus has been demonstrated through genome mining approaches [4–6], revealing many biosynthetic gene clusters (BGCs) encoding the production of small molecules, also known as natural products. Despite being one of the most extensively studied genera from a natural products’ standpoint, only an estimated 3% of their biosynthetic potential has been experimentally characterized [6]. Public deposition of sequenced genomes has accelerated the discovery of BGCs thanks to the development of genome mining tools such as antiSMASH [7], community efforts to annotate BGCs through initiatives such as MiBIG [8], and resources to link molecules to genes, such as the Paired Omics Data Platform [9]. However, public deposition of curated microbial metabolomes or metabolomics datasets, which could help to confirm the production of such molecules, are just starting [10, 11]. Although thousands of microbial molecules can be found in databases, such as NPAtlas [12] or Dictionary of Natural Products [13] only a few contain reference spectral data such as tandem mass spectrometry (MS/MS).

Several strategies are used to characterize the biosynthetic potential of microorganisms. On one hand, rapid advances in sequencing technologies coupled to genome mining tools have contributed to the understanding of the biosynthetic potential of microorganisms and even made possible analysis at the pangenome scale [14]. However, limitations of these tools - due to inaccurate, incomplete or even lack of training data for developing new algorithms - can restrict the advance of knowledge. On the other hand, experimental data to validate the products of such BGCs is limited. Classical approaches involving culturing, extraction, isolation, purification and structural characterization are time consuming and expensive, however they have generated reliable data to confirm the expression of reported BGCs. These culture-based approaches involve testing different conditions on a single microbial strain [15], leading to discovering optimal factors that trigger the expression of silenced BGCs [16], further increasing interest in more in-depth biosynthetic studies.

Besides the advances in computational tools and resources available for metabolomics analysis, the lack of public metabolomics datasets remains a bottleneck to experimentally demonstrate the biosynthetic potential of microorganisms. Development of tools for predicting and comparing experimental data, as well as reference databases, has only been possible due to the availability of such public data. Additionally, well-characterized datasets are fundamental for further development in machine learning algorithms and benchmarking tools [17]. Such datasets will enable the scientific community to further advance computational approaches, tools and resources while validating the biosynthetic potential of microorganisms.

Due to the evident need for publicly available microbial metabolomes and as a resource to assess the discovery potential of small molecules, we provide metabolomics datasets for 948 microbial strains. We cultured 440 strains of *Actinomycetes* in five culture media to increase the success in production and detection of natural products. We expanded this analysis to include an additional 508 bacterial isolates cultured under a single condition (that also includes non-*Actinomycetes*, Supplementary Table S1), in order to increase the chemical diversity. Crude extracts were analyzed using untargeted liquid chromatography coupled to tandem mass spectrometry (LC-MS/MS). From these datasets, 260,488 out of the ~2 million detected MS/MS (~13.3% corresponding to 2352 molecules) were annotated using public spectral libraries. Therefore, this dataset serves as a valuable resource for microbial molecules data mining as demonstrated by detection of recently discovered non-ribosomal peptides and analogues.

## Results

### Detecting microbial molecules from 948 bacterial strains

Multiple factors influence the production of microbial small molecules. Strategies optimizing growth conditions and small molecules’ production continue to evolve as our understanding of the environmental and chemical cues that influence microbial metabolism remains incomplete [18, 19]. Most strains in this study were sourced from a microbial collection with known optimal growth conditions. Therefore, we selected five complex media, including at least one optimal media for growth (see *Materials and Methods* section), to increase the success of detecting biosynthetic products [15].

**Fig. 1:**
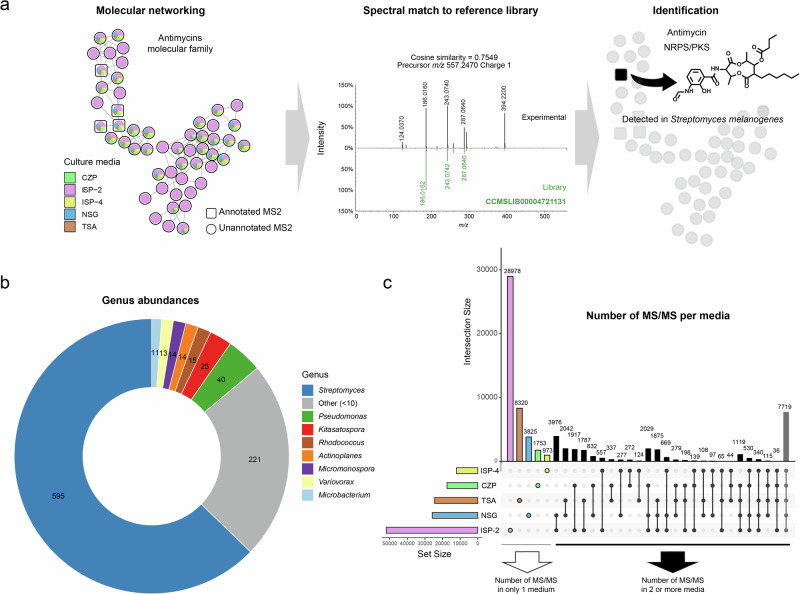
Production of small molecules from 948 bacterial strains. **a** Molecular networking approach provides a visualization of detected molecules (nodes represent detected molecules based on their fragmentation spectra MS/MS and connected based on spectral similarity. Color map indicates the culture media used in the study. Pie charts in the molecular family represent the proportion of fragmentation spectra MS/MS per media). Spectral match to reference library is a step performed after the molecular networking approach where each fragmentation spectra is compared against spectral libraries. Matches to reference spectra accelerate the identification of molecules. The mirror plot comparison of fragmentation spectra corresponds to antimycin, a depsipeptide assembled from a hybrid non-ribosomal peptide synthetase (NRPS) and polyketide synthase (PKS) biosynthetic pathway. Experimental MS/MS (top, black) and reference spectra from GNPS spectral libraries (bottom, green). This identification allows to annotate the molecular family as antimycins as connected nodes share chemical relationships based on spectral similarity; **b** Diversity of the bacterial strains based on genus. *Streptomyces* genus forms the highest proportion of the selected strains for this study. The “Other (<10)” category refers to genera with fewer than 10 strains. This category comprises 221 strains, belonging to 102 genera; **c** Upset plot showing the number of fragmentation spectra (MS/MS) per culture condition (culture media). In this visualization, the Intersection Size indicates the number of MS/MS that are unique (colored according to each culture condition), shared between two or more culture conditions (black) and shared among all culture media (grey). These are shown in the matrix and in the top bar-plot. The Set Size visualized as the left bar-plot corresponds to the total number of fragmentation spectra (MS/MS) per individual culture condition, regardless of their overlap with other culture conditions. These are shown in the matrix and in the left bar-plot. In short, the Set Size bars indicate how many MS/MS are per culture condition individually, while the intersection bars indicate how many MS/MS are in overlap with other culture media

Although there is a trade-off between microbial growth and detection of microbial molecules due to the interference of complex media and compatibility with mass spectrometry [18], the lack of available data and reference molecules to facilitate identification slows the discovery process. To expand the number of microbial molecules with available MS/MS data, we first studied the metabolome of 440 *Actinomycetes* using five culture media (Supplementary Fig. S1) and applied the molecular networking approach [20, 21]. Briefly, as shown in Fig. 1a, fragmentation spectra (MS/MS) are represented by nodes, connected based on spectral similarity and matched against spectral libraries for identification [21]. The number of MS/MS reflects the diversity of ion species, including adducts, in source fragments, multimeric species, rather than unique molecules [22–35]. However, a higher number of observed MS/MS spectra under a given culturing condition increases the likelihood of capturing a greater diversity of unique molecules. Therefore we use the number of MS/MS spectra as a proxy to assess culturing conditions that maximize the number of observed molecules.

Among the five media used, ISP-2 (International *Streptomyces* Project medium 2) [36] has been commonly used for the growth, production and detection of small molecules [18, 36]. Additionally, ISP-2 medium has shown compatibility with mass spectrometry approaches without producing high background or interference [37]. We observed that for some molecular families, such as the depsipeptides antimycins [38], most of the MS/MS were present in this media (Fig. 1a), indicating that ISP-2 still supported the detection of a considerable number of molecules (Supplementary Fig. S1). Therefore, due to ISP-2 being commonly and successfully used for both growing and production of microbial molecules, we included in the analysis additional isolates mostly corresponding to the genus *Streptomyces* (Fig. 1b) cultured using this medium. It is important to highlight that a high number of unique MS/MS were detected from each medium, including ISP-4, which was the medium with the lowest number of unique MS/MS (973 spectra) (Fig. 1c). This is consistent with the OSMAC strategy [15], in which variation of growing conditions, such as culture media, has a direct effect on the levels of production and diversity of small molecules. Additionally, a substantial number of detected MS/MS were shared across all five media (7719 detected MS/MS, Fig. 1c), highlighting their suitability for microbial molecule production. The annotation rate (~13.3%) of the data generated through LC-MS/MS suggests significant potential for discovery of microbial natural products. We employed untargeted LC-MS/MS data to overcome the potential interference of ion suppression observed using direct infusion methods without chromatography to reduce chemical complexity during detection by MS. The metabolomics data is publicly available to facilitate future discovery of microbial molecules using spectral searches within the GNPS environment or using other tools including MZmine and MS-DIAL [39, 40].

### Annotating the data

The entire dataset of 948 strains contains a total of 1,947,363 fragmentation spectra that represent 105,499 unique MS/MS spectra after MS-Cluster merging. Molecules from the culture media were removed by using exclusion lists during data acquisition (see Materials and Methods section), while signals from noise were filtered out during molecular networking using blank samples (see Materials and Methods section, Molecular Networking settings). Although the number of natural products will be far lower since one molecule might be found in many different ion forms due to in-source fragmentation, different adducts or multimeric species, this represents the upper bound of the discovery potential of candidate molecules that is present in this data set. This is important, as different researchers might be able to access different instruments and methods for LC-MS/MS, and detection of molecules as different species might facilitate the chances of discovery of molecules within these datasets. We used existing public MS/MS libraries in the Global Natural Products Social molecular networking ecosystem (GNPS) to provide annotations [20, 21]. This resource includes more than 30 different public reference libraries (listed in the Materials and Methods section).

GNPS also has propagated libraries [41]. These are libraries where the MS/MS is annotated based on similarity to another annotated MS/MS spectrum. This approach enables the discovery of analogs and metabolized versions of related molecules. When combining experimental and propagated libraries [41], we were able to double the number of annotated spectra from 1235 (130,317 MS/MS spectra) to 2352 (260,488 MS/MS spectra, see networking links provided in the Materials and Methods section). It should be noted that not every annotation will be correct. The annotation settings used give rise to ~1% false discovery rates [42].

Examples of annotated MS/MS spectra (Fig. 1) include the antimycin antibiotics. These molecules are depsipeptides assembled from a hybrid non-ribosomal peptide synthetase (NRPS)/polyketide synthase (PKS) biosynthetic pathway encoded by *Streptomyces* genus [43, 44]. Information regarding geographical distribution of the *Streptomyces* isolates used in this study enabled to confirm previous findings indicating a worldwide distribution of antimycin BGCs [44]. Other examples of bioactive detected molecules include actinomycins, antibiotic and anticancer compounds [45–48]; pteridic acid, a polyketide produced by *Streptomyces* and responsible for plant resistance to abiotic stress [49]; sevadicin, a NRP previously reported from *Paenibacillus larvae* [50]; staurosporine, an apoptosis inducer alkaloid originally isolated from *Streptomyces* [51, 52]; trienomycin, antibiotic with anticancer potential [53, 54]; spiramycin, a long time used macrolide antibiotic in humans originally isolated from soil *Streptomyces* [55]; josamycin (also known as leucomycin A3, kitasamycin A3, turimycin A5) a macrolide antibiotic with antiviral properties against influenza [56]; the anticancer agent daunorubicin, a member of the type II polyketide family [57]; the immunosuppressant agent tacrolimus (also known as FK-506), a polyketide macrolide more potent than cyclosporin [58, 59]; and, siderophores, such as desferrioxamine [60], a chelating agent used to remove excess metals with therapeutic applications for humans (e.g., desferrioxamine B, commercially known as Desferal) [61]. After manual curation of the experimental data, we provided 1215 annotated spectra corresponding to 698 non-redundant microbial molecules (Supplementary Table S2) created from classical molecular networking and library annotations in GNPS2 (link provided in Materials and Methods section). We expanded the annotations to 2352 by using propagation libraries [41]. Additionally, we created a database of 515 known molecules from *Streptomyces* (Supplementary Table S3), their fragmentation spectra were predicted and compared against the experimental data acquired for this study. Then, manual verification enabled us to confirm 116 molecules (Supplementary Table S4), providing additional annotations to the ones obtained through the GNPS public libraries. The database corresponding to the 515 known molecules from *Streptomyces* is available as Supplementary Table S3, which can be used to propagate annotations [62, 63], to match experimental data of molecules that have not been deposited in public libraries of experimental data until now. The curated annotations were uploaded to the Collaborative Microbial Metabolite Center CMMC (https://cmmc.gnps2.org/) and are publicly available.

This resource adds reference spectra for more than a thousand known molecules, including membrane lipids and other common natural product metabolites, such as those nutrients (e.g., amino acids) present in culture media. Then, even if no annotation is provided, its connection to other similar spectra within a molecular family increases the probability of annotation at the molecular family (e.g., propagated annotation) as shown for antimycins in Fig. 1a. Our work increases the experimental evidence of the Actinomycetota metabolome, mostly from *Streptomyces* species. Therefore, we provided proof of detection and curated annotations for more than thousand microbial molecules produced by the selected bacteria strains included in this study.

### Estimating the discovery potential

**Fig. 2:**
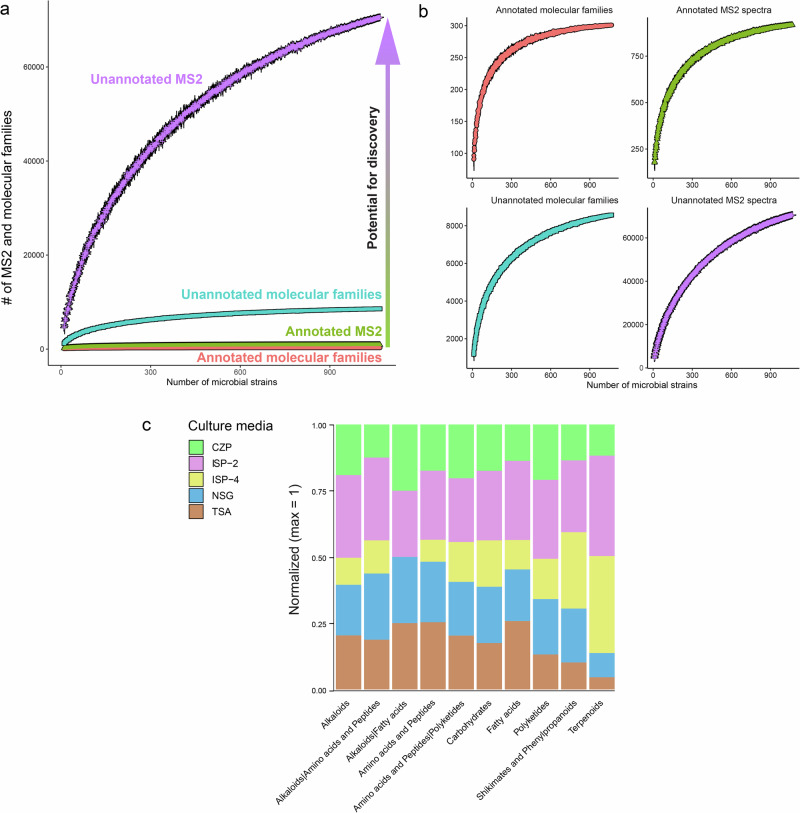
Chemical space detected from microbial strains in this study. **a** Rarefaction curve displaying the number of strains (x-axis) against the number of MS/MS spectra and molecular families (y-axis); **b** Zoom in of rarefaction curves by annotated molecular families, annotated MS/MS spectra, unannotated molecular families and unannotated MS/MS spectra. Color code as shown in the figures; **c** Number of annotated spectra using GNPS spectral libraries and classified by biosynthetic pathways (NPclassifier) [65]. Bar plots showing the variation of the annotated molecules by five of the culture media. The number of annotations as shown in Fig. 1 (main text) was normalized to 1 as maximum by biosynthetic pathway. Briefly, the number of annotated MS/MS was calculated per culture condition and per biosynthetic pathway and normalized to the maximum within each pathway. Color code as shown in the figure per culture condition

To estimate the discovery potential, we used a rarefaction approach (Fig. 2a, b, see Methods for details). Using rarefaction, each MS/MS spectrum in the data of a sample is counted, then for the next sample each additional MS/MS that is not found in the data from the first sample is counted, and then for the third sample, data will be added but only if the MS/MS is not found in the first two samples. Rarefaction is continued in this fashion until all the MS/MS are found in the data from the 948 bacterial samples (3439 files). This can be performed after merging all identical spectra (consensus spectra) and also at the molecular family level (connected nodes). This analysis demonstrates that most MS/MS spectra remain unannotated. However, by grouping these spectra into molecular families, the annotation gap is reduced to provide a higher level of the detected chemical diversity and potential of discovery. The output of GNPS based molecular networking, which includes a clustering step performed by MS-Cluster [21, 64], was used to perform the rarefaction analysis. The rarefaction analysis reveals that the number of annotated molecular families is 328 while the unannotated families are reaching above 8000, highlighting that there is a vast number of molecular families that remain unannotated. This includes unannotated families of different ion forms of the annotated molecular families where the MS/MS does not share sufficient similarity to known ions. As molecular networking is complemented with search against spectral libraries and further chemical classification is possible, additional evidence regarding a chemical diversity approach was provided (Fig. 2c, Supplementary Fig. S2) based on chemical classification using NPClassifier [65]. To facilitate the comparison across different culture conditions, the number of annotated spectra was normalized per culture condition and biosynthetic pathway (Fig. 2c). It was observed that ISP-2 provided a more consistent detection of molecules across the biosynthetic pathways and in some cases, relatively higher. This was evident when comparing ISP-2 and TSA regarding the biosynthetic pathway of terpenoids. In other cases, for instance when focusing on ISP-4, low proportion of annotations belonging to Alkaloids and Alkaloids|Amino acids and Peptides were observed, while no detection of molecules belonging to Alkaloids|Fatty acids pathway was observed. Altogether, these observations further supported the inclusion of additional datasets corresponding to microbial extracts obtained in ISP-2 to increase the coverage of detected molecules from this study. This is consistent with other reports indicating high antimicrobial activity of *Streptomyces* strains when cultured on ISP-2 media [66, 67].

**Fig. 3:**
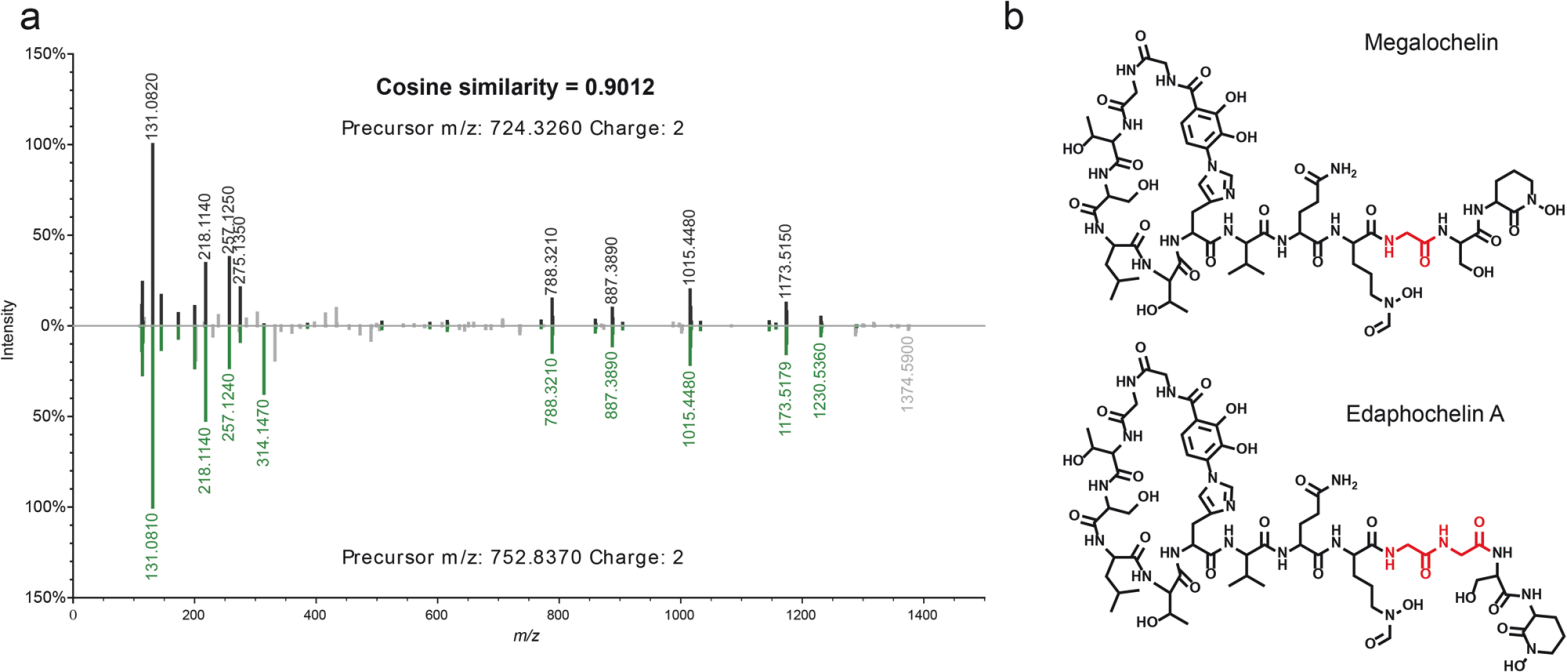
Recently discovered siderophores from *Streptomyces* sp. **a** Mirror comparison of MS/MS spectra of megalochelin [68] (top) and edaphochelin A (Behsaz et al. submitted) (bottom); **b** Chemical structures of megalochelin (top) and edaphochelin A (bottom). The location of the additional glycine residue predicted by ModiFinder [69] (Supplementary Fig. S3) is consistent with the chemical structure of edaphochelin A

As a demonstration of the potential of discovery that the datasets provided in this work contain, a recently discovered NRP siderophore related to megalochelin [68] was detected. This analogue of megalochelin was also detected from a *Streptomyces* strain and identified as edaphochelin A (Behsaz et al., submitted). Since both molecules shared a chemical similarity based on their fragmentation pattern (Fig. 3), the chemical structure of megalochelin was used to confirm such similarity and more importantly, the location of the chemical modification observed in edaphochelin A. By using ModiFinder [69], a recently developed tool able to predict the location of a chemical modification of an unknown molecules based on its spectral similarity when compared against a known molecule, an additional glycine residue was predicted to be present in edaphochelin A (Supplementary Fig. S3). The prediction provided by ModiFinder is consistent with the chemical structure proposed for edaphochelin A and a remarkable example that showcases the use of metabolomics datasets of this kind and application of computational developments to further expand the discovery of microbial molecules present in public repositories.

## Discussion

Several gaps still exist in the search for new microbial small molecules. While tens of thousands of microbial molecules have been discovered to date [12], the genetic information, in the form of gene clusters, encoded in microbial genomes suggests a potential exceeding millions [6]. However, genetic potential does not guarantee production under laboratory conditions. Therefore, detecting the products of such genomic potentials opens several avenues for investigating the roles of such molecules mediating interkingdom relationships, such as plant-microbe interactions (e.g., pteridic acids reverse drought and salinity stress in plants) [49]. In addition, further biosynthetic studies are facilitated once a gene product has been detected to demonstrate the geographical distribution and evolution of BGCs (e.g., pteridic acids and antimycins BGCs distribution) [44, 49]. The discovery of BGCs is expected, and we envision many of the detected molecules in our datasets will be linked to such microbial genes. A recently discovered group of NRP siderophores, megalochelins [68], that show a characteristic fragmentation pattern also shared by edaphochelins (Behsaz et al., submitted) (Fig. 3) highlight the value of publicly available experimental microbial metabolomics datasets such as the one provided in this study to accelerate the discovery and link to BGCs of microbial producers. By considering estimates, where it was suggested that one *Streptomyces* genome may contain between 8–83 BGCs [70], and could produce 100,000 molecules [71, 72], access to public datasets for mining microbial molecules is necessary. Since a single BGC can be involved in the biosynthesis of multiple compounds [73], the number of microbial molecules is expected to far exceed the number of BGCs present in a genome.

Current strategies for identifying microbial molecules rely on publicly available chemical information in spectral libraries and databases. By using tandem mass spectrometry, the need for reference spectra to assist the dereplication steps is evident. However, the lack of such references causes delays in the discovery of new molecules, a key point in the search for new antibiotics and other bioactive molecules. This is one of the main reasons for parallel strategies by propagating annotation of tandem mass spectrometry data [62, 74, 75]. For instance, the recent discovery of angucycline analogues [76], one of the largest families of polyketides produced by *Streptomyces*, was possible due to the annotation of detected spectra from previous work [77], and their availability in public libraries, such as GNPS spectral libraries. To accelerate the discovery of new microbial molecules, efforts to increase the amount of reference spectra must be made.

In addition to providing a large and curated microbial reference dataset, we have made these datasets public and accessible as part of community resources via the GNPS platform and the Mass Spectrometry Interactive Virtual Environment (MassIVE) repository (See Data availability section). These datasets have also been integrated in microbeMASST [78], a novel MS/MS search tool, representing an evolution of the original MASST [79], that allows searching for single MS/MS spectra against a reference microbial metabolomics database. With this tool, researchers can search unknown fragmentation spectra and gain information about microbial producers, confirming or generating biosynthetic hypotheses. Furthermore, we have provided curated annotations as part of the Collaborative Microbial Metabolite Center CMMC (https://cmmc.gnps2.org/). This large dataset is also part of the community effort to enable metabolomic analysis at the repository scale via ReDU (https://redu.gnps2.org/) [80, 81]. In line with the need for data standardization, which includes growth media and culture conditions [82], this resource supports a wide range of applications, from identifying small molecule producers to benchmarking annotation tools and improving predictive algorithms for linking molecules to genes.

## Conclusions

We provided tandem mass spectrometry data from hundreds of bacterial strains, primarily from *Streptomyces* genus. We provided 1215 annotated spectra corresponding to 698 non-redundant microbial molecules in addition to 116 annotations based on propagated annotations that had not been annotated yet in public mass spectrometry libraries. Our results will support future research focused on investigating the ecological role of thousands of molecules detected in our datasets, even though it is well known that many BGCs are silent or cryptic under laboratory conditions. Additionally, many more predicted BGCs within other uncovered microbial groups are likely to encode the reported produced compounds with relevant ecological and pharmacological activities. Finally, we envision this large dataset of microbial molecules and associated information as a valuable resource, not just for dereplication purposes but also, to identify unknown producers of known bioactive molecules ultimately facilitating the linkage of molecules to their biosynthetic genes.

### Limitations/scope

The detected molecules are limited to the culture conditions, extractions, liquid chromatography and mass spectrometry settings used during the data acquisition. Several fragmentation spectra might correspond to adducts, in source fragments and multimers belonging to the same molecule, so redundancy is expected in the data set. Yet, despite those caveats, it shows there is a lot of discovery potential. Annotation of fragmentation spectra enables us to begin to tackle this redundancy, but annotation rates are still limited to available spectral libraries as stated in this study.

## Materials and methods

### Materials

*Actinomycetes* strains were obtained from the Agricultural Research Service Culture Collection (NRRL) https://nrrl.ncaur.usda.gov/.

Nunc™ 96-Well Polypropylene DeepWell™ storage plates, catalog number: 278743; acetonitrile with formic acid 0.1% (v/v) LC-MS grade (Optima, Fisher Chemical) catalog no. LS120-4; water with formic acid 0.1% (v/v) LC-MS grade (Optima, Fisher Chemical) catalog no. LS118-4.

#### Actinomycetes strains

Microorganisms received from the provider as freeze dried pellets were resuspended in 50% glycerol and transfer to 96 deep well plates for inoculation of media in 96-DeepWell plates and stored under −80 °C until microbial cultures were initiated.

### Media

All media were prepared according to the provider recommendations and recipes available through the NRRL (https://nrrl.ncaur.usda.gov/) for each *Actinomycetes* strain: International *Streptomyces* Project Yeast Extract-Malt Extract agar (ISP-2 agar) (Difco No. 277010); International *Streptomyces* Project Synthetic Salts-Starch Medium (ISP-4 agar) (Difco No. 277210); Trypticase Soy Agar (TSA) (Difco No. 211768); N-Z Amine with Soluble Starch and Glucose agar (NSG agar) (Sigma Chemical Company Catalog No. C 0626); Czapek’s Solution and agar (CZP agar) (Sigma Aldrich Catalog No. C 6095).

### Culture and incubation

Cultures of microbial strains were performed as follows: from glycerol stocks, an inoculum of 20 uL of glycerol stocks were transferred into 96-deep well plates containing 1 mL of solid media and incubated at 28 °C during 14 days.

### Extraction

After a 14-day incubation period, plates were placed under −80 °C until extraction procedure. Three freeze–thaw cycles of 10 min each were performed before extraction steps. For agar-based media cultures, 1 mL of methanol was added to each well, and plates were submitted to sonication for 15 min (Branson 5510, Marshall Scientific, Hampton, NH, USA), centrifugation for 15 min at 2000 RPM (865 × *g*) (Sorvall Legend RT, Marshall Scientific, Hampton, NH, USA), transferring of supernatant to a clean 96-well plate and dried out in a centrifugal vacuum concentrator, Centrivap (Labconco, Kansas City, MO, USA). Samples were dissolved in 500 μL 50% methanol:water LC-MS grade, and 100 μL were transferred to a clean 96-well plate and dried out in the centrifugal vacuum concentrator.

### LC-MS/MS acquisition

Obtained extracts were dissolved in 200 μL 50% methanol_(aq)_ containing 1 μM sulfadimethoxine as internal standard for LC-MS monitoring. Untargeted LC-MS/MS acquisition was performed on a Vanquish Ultrahigh Performance Liquid Chromatography (UPLC) system coupled to a Q-Exactive^TM^ Hybrid Quadrupole-Orbitrap^TM^ (Thermo Fisher Scientific, Bremen, Germany). Chromatographic separation was performed on a Kinetex 1.7 μm 100 Å pore size C18 reversed phase UHPLC column 50 × 2.1 mm (Phenomenex, Torrance, CA) with a constant flow rate of 0.5 mL/min. The following solvents were used during the LC-MS/MS acquisition: water with 0.1% formic acid (v/v), Optima™ LC/MS Grade, Thermo Scientific™ (solvent A) and acetonitrile with 0.1% formic Acid (v/v), Optima™ LC/MS Grade, Thermo Scientific™ (solvent B). After injection of 2 μL of sample into the LC system, the elution was performed isocratically with 5% B from 0 to 0.5 min, then with a multistep linear gradient from 5 to 50% B (0.5–6 min), 50 to 99% B (6–8 min), 99% B (8–10 min), 99 to 5% B (10–10.5 min), and 5% B (10.5–12 min). Data dependent acquisition (DDA) mode was used for acquisition of tandem MS (MS/MS) data with a default charge state of 1. An exclusion list was generated for each culture media and used as input for untargeted LC-MS/MS in data dependent acquisition mode. The exclusion list was created using a Python script available in: https://github.com/lfnothias/IODA_MassSpec [83]. Full MS was acquired using 1 microscan at a resolution (R) of 35,000 at *m/z* 200, automatic gain control (ACG) target 5e5, maximum injection time (IT) of 100 ms, scan range *m/z* 250–3750 and data acquired in profile mode. DDA of MS/MS was acquired using 1 microscan at a resolution (R) of 35,000 at *m/z* 200, automatic gain control (ACG) target 5e5, top 5 ions selected for MS/MS with isolation window of *m/z* 2.0 with scan range *m/z* 200–2000, fixed first mass of *m/z* 100 and stepped normalized collision energy (NCE) of 20, 25 and 30 eV, minimum ACG target 2.50e4, intensity threshold 2.5e5, apex trigger 2–15 s, all multiple charges included, isotopes were excluded and a dynamic exclusion window of 5 s. Analytical blanks and mixture of sulfamethazine, sulfamethizole, sulfachloropyridazine, sulfadimethoxine, amitriptyline, and coumarin-314 (10 μM) were injected after every 96 samples as quality control for monitoring instrument (LC-MS) performance.

### Molecular networking

A molecular network was created using the online workflow (https://ccms-ucsd.github.io/GNPSDocumentation/) on the GNPS website (http://gnps.ucsd.edu) [20]. The data was filtered by removing all MS/MS fragment ions within +/−17 Da of the precursor m/z. MS/MS spectra were window filtered by choosing only the top 6 fragment ions in the +/−50 Da window throughout the spectrum. The precursor ion mass tolerance was set to 0.02 Da and a MS/MS fragment ion tolerance of 0.02 Da. A network was then created where edges were filtered to have a cosine score above 0.7 and more than 6 matched peaks. Further, edges between two nodes were kept in the network if and only if each of the nodes appeared in each other’s respective top 10 most similar nodes. Finally, the maximum size of a molecular family was set to 100, and the lowest scoring edges were removed from molecular families until the molecular family size was below this threshold. The spectra in the network were then searched against GNPS’ spectral libraries. The library spectra were filtered in the same manner as the input data. All matches kept between network spectra and library spectra were required to have a score above 0.7 and at least 6 matched peaks.

The molecular networking analysis with metadata including culture media can be accessed through the link: https://gnps.ucsd.edu/ProteoSAFe/status.jsp?task=aa06c41deb25441b818f5e716dd1d095.

The molecular networking analysis with additional metadata including culture media without removing MS/MS from blanks can be accessed through the link: https://gnps.ucsd.edu/ProteoSAFe/status.jsp?task=f69176f25e3d4aed93f335b05b8a5fba.

The molecular networking analysis for 440 *Actinomycetes* with additional metadata including culture media without removing MS/MS from blanks (blanks already removed using exclusion list during LC-MS/MS acquisition) can be accessed through the link: https://gnps.ucsd.edu/ProteoSAFe/status.jsp?task=cb82ed06161046f5b9cd9f8638374eea.

Molecular networking was also created using the GNPS2 platform and maintaining the same settings described above. This facilitated the visualization of the large dataset composed of 4020 files, 593 corresponding to blanks and quality controls used to monitor instrument performance during LC-MS/MS acquisition. The molecular networking analysis can be accessed through the following link (this job is used for Supplementary Table S2): https://gnps2.org/status?task=e4a5b96dddfd4ccb8f7ab54095684e10.

### In silico annotation via network annotation propagation

Network annotation propagation (NAP) [62] was performed on a subset of the dataset (Classical molecular networking job link: https://gnps.ucsd.edu/ProteoSAFe/status.jsp?task=0ea632474af24eb6a84e576d0aa585ca) due to memory limitations (running NAP on entire dataset is not currently possible, but improvement efforts are ongoing via ChemWalker approach [63]), via the GNPS platform using a customized database containing 515 reported products of *Streptomyces* not yet available through GNPS libraries (Supplementary Table S3). The parameters used the 10 first candidates for consensus score, 15 ppm accuracy for exact mass candidate search, positive acquisition mode, and 0.5 cosine value to subselect inside a cluster. Fusion results were used to determine a consensus, searching only for [M + H]^+^ adduct type. A maximum of 10 candidate structures were used in the graph. The following databases were searched: Dictionary of Natural Products (DNP) [13], Super Natural II [84], GNPS [20], and Chemical Entities of Biological Interest (ChEBI) [85]. GNPS NAP job link: https://proteomics2.ucsd.edu/ProteoSAFe/status.jsp?task=b89434115a5248d08b92ecb6d6a719d1.

### Propagated annotation via suspects library

In addition to the spectral libraries included in the molecular networking analysis, propagated annotations were obtained on the same datasets using the suspects libraries [41]. The molecular networking analysis including the suspects library can be accessed through the link: https://gnps.ucsd.edu/ProteoSAFe/status.jsp?task=22521ea20bf5412a819124fac1db0efb.

The following link provides access to the molecular networking analysis and library search with all libraries including propagated (e.g., suspects library) in the GNPS2 platform: https://gnps2.org/status?task=d6a0bab81cd844bb8cddccef17764a32.

## Supplementary information

Revised Supplementary Information

Supplementary Table S1

Supplementary Table S2

Supplementary Table S3

Supplementary Table S4

## Data Availability

All the datasets used and generated for this study have been deposited online in the public repository Mass Spectrometry Interactive Virtual Environment (MassIVE). Bacterial strains were provided by the NRRL Agricultural Research Service Culture Collection from the U. S. Department of Agriculture (USDA) and RIKEN BioResource Research Center. Public genome data from all bacterial strains used in this study are available in the NCBI genome database. The following mass spectrometry datasets can be accessed through the MassIVE repository at https://massive.ucsd.edu/: MSV000088235 GNPS - 100 *Actinobacteria* strains in ISP-2 media; MSV000088742 GNPS - 95 *Actinobacteria* strains in ISP-2 liquid media; MSV000088196 GNPS - Populus trees 153 microbial isolates; MSV000088763 GNPS - *Actinobacteria* cultured in ISP-2 agar media; MSV000088764 GNPS - *Actinobacteria* cultured in NSG agar media; MSV000088800 GNPS - *Actinobacteria* cultured in Czapek agar media; MSV000088801 GNPS - *Actinobacteria* cultured in TSA media; MSV000088816 GNPS - *Actinobacteria* cultured in ISP-4 media; MSV000089090 GNPS - 95 *Actinobacteria* strains in ISP-2 liquid media; MSV000089742 GNPS - 95 *Actinobacteria* strains in ISP-2 liquid media; MSV000089813 GNPS - *Actinobacteria* cultured in ISP-2 agar media; MSV000089815 GNPS - *Actinobacteria* cultured in NSG agar media; MSV000089816 GNPS - *Actinobacteria* cultured in Czapek agar media; MSV000089817 GNPS - *Actinobacteria* cultured in TSA media; MSV000089818 GNPS - *Actinobacteria* cultured in ISP-4 media.
