# Supplementary material for: The undiscovered natural product potential of Actinomycetes
Source: J Antibiot (Tokyo). 2025 Dec 2;79(2):80–92. doi: 10.1038/s41429-025-00876-x (PMC12834688; doi:10.1038/s41429-025-00876-x)
Supplement: Supplementary file 1 — Revised Supplementary Information [file 41429_2025_876_MOESM1_ESM.docx]

**Supplementary information**

**The Undiscovered Natural Product Potential of *Actinomycetes***

Andrés M. Caraballo-Rodríguez^1,2^, Andrés Cumsille^3,4^, Sarolt Magyari^5^, Maria Taboada-Alquerque^6^, Bahar Behsaz^7^, Tiago F. Leão^8^, Kirk Broders^9^, Yasin El Abiead^1^, Jason A. Clement^10^, Vincent Charron-Lamoureux^1^, Simone Zuffa^1^, Louis-Félix Nothias^11,12^, Mengzhou Hu^1^, Christopher Leone^1^, Sarvar A. Kakhkhorov^1,13^, Beatriz Cámara^3^, Hosein Mohimani^14,15^, Pieter C. Dorrestein^1,2,16,17^

^1^Skaggs School of Pharmacy and Pharmaceutical Sciences, University of California San Diego, 9500 Gilman Drive, San Diego, California, 92093-0751, United States, ^2^Collaborative Mass Spectrometry Innovation Center, Skaggs School of Pharmacy and Pharmaceutical Sciences, University of California San Diego, 9500 Gilman Drive, San Diego, California, 92093-0751, United States, ^3^Departamento de Química y Centro de Biotecnología Daniel Alkalay Lowitt, Laboratorio de Microbiología Molecular y Biotecnología Ambiental, Universidad Técnica Federico Santa María, Valparaíso, 2340000, Chile, ^4^Department of Plant Pathology and Wisconsin Institute for Discovery, University of Wisconsin-Madison, Madison, Wisconsin, 53703, United States, ^5^Institute of Microbiology, Eidgenössische Technische Hochschule (ETH) Zürich, Vladimir-Prelog-Weg 4, 8093 Zürich, Switzerland, ^6^School of Pharmaceutical Sciences, University of Cartagena, Cartagena, Colombia, ^7^Carnegie Melon University & Chemia Biosciences Inc, ^8^Núcleo de Bioensaios, Biossíntese e Ecofisiologia de Produtos Naturais (NuBBE), Institute of Chemistry, São Paulo State University (UNESP), Araraquara, São Paulo 14800-901, Brazil, ^9^USDA, Agricultural Research Service, National Center for Agricultural Utilization Research, Mycotoxin Prevention and Applied Microbiology Research Unit, 1815 N. University, Peoria, IL, 61604, United States, ^10^Baruch S. Blumberg Institute, 3805 Old Easton Rd, Doylestown, Pennsylvania, 18902, United States, ^11^Institut de Chimie de Nice, Université Côte d'Azur, CNRS, Nice, France, ^12^Interdisciplinary Institute for Artificial Intelligence (3iA), Côte d'Azur, Sophia-Antipolis, France, ^13^Laboratory of Physical and Chemical Methods of Research, Center for Advanced Technologies, Tashkent 100174, Uzbekistan, ^14^Carnegie Melon University, ^15^Department of Computational Medicine, University of California Los Angeles, Los Angeles, California, 90095, United States, ^16^Department of Pharmacology, University of California San Diego, 9500 Gilman Drive, San Diego, California, 92093-0751, United States, ^17^Center for Microbiome Innovation, University of California San Diego, 9500 Gilman Drive, San Diego, California, 92093-0751, United States

Supplementary information is available at the Journal of Antibiotics’ website and includes the following tables and figures:

**Supplementary Table S1**. GNPS2 complete metadata for 948 strains

**Supplementary Table S2.** CMMC formatted table of annotated spectra

**Supplementary Table S3.** Customized database of *Streptomyces* molecules formatted for NAP

**Supplementary Table S4.** CMMC formatted table of in silico predicted and matched to experimental MS2

**Supplementary Figure S1.** Differential production of small molecules by *Actinomycetes* strains

**Supplementary Figure S2.** Number of annotated spectra using GNPS spectral libraries and classified by biosynthetic pathways

**Supplementary Figure S3.** Prediction of modification site of recently discovered siderophores from *Streptomyces* sp.


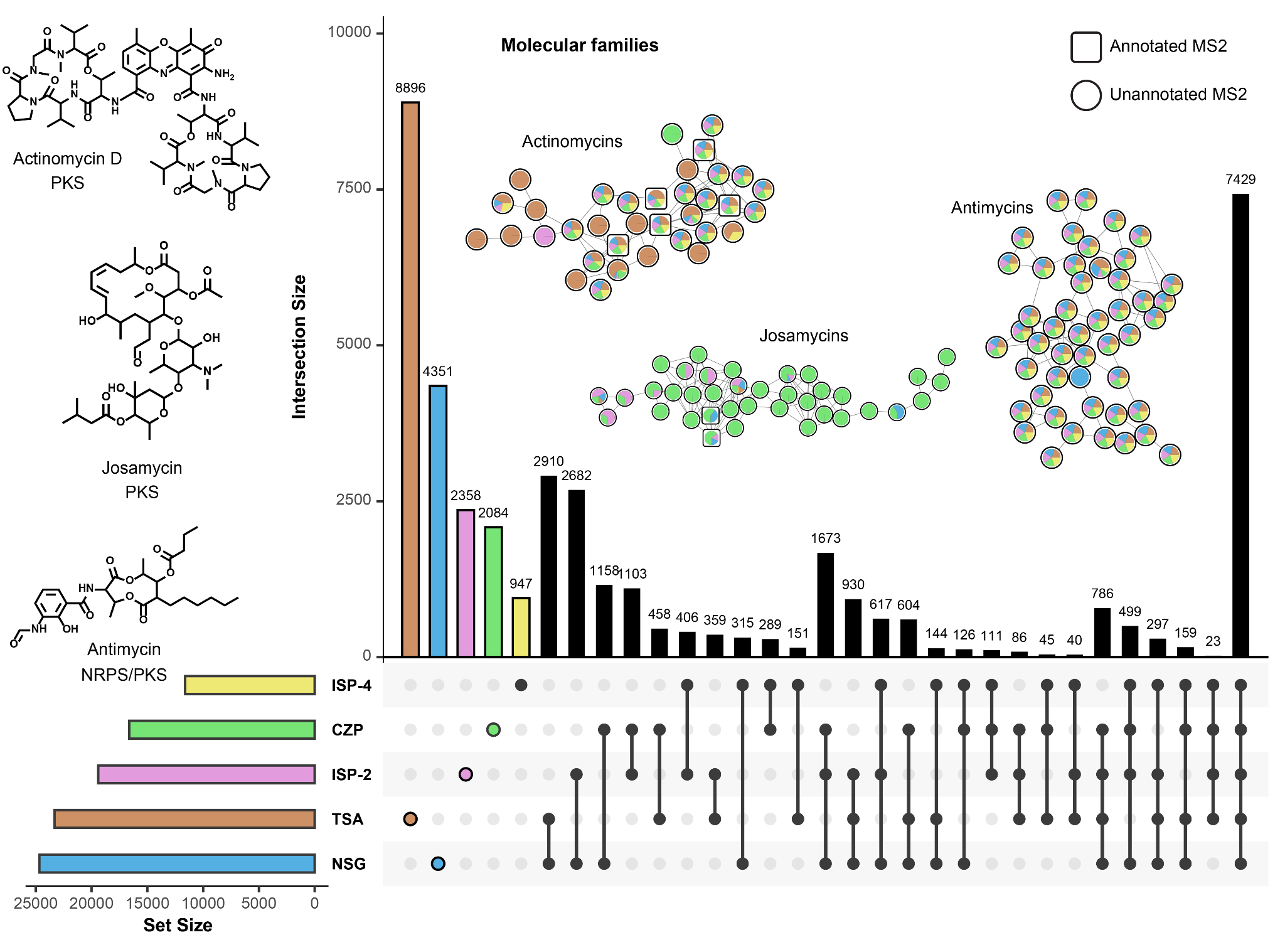


**Supplementary Figure S1.** Differential production of small molecules by *Actinomycetes* strains. **UpSet plot:** Datasets corresponding to a subset of 440 actinomycete strains cultured in five different media conditions. In this visualization, the Intersection Size indicates the number of MS/MS that are unique (colored according to each culture condition), shared between two or more culture conditions and shared among all culture media (black). These are shown in the matrix and in the top bar-plot. The Set Size visualized as the left bar-plot corresponds to the total number of fragmentation spectra (MS/MS) per individual culture condition, regardless of their overlap with other culture conditions. These are shown in the matrix and in the left bar-plot. In short, the Set Size bars indicate how many MS/MS are per culture condition individually, while the intersection bars indicate how many MS/MS are in overlap with other culture media. **Molecular families** created by applying the molecular networking approach which provides a visualization of detected molecules. Nodes represent detected molecules based on their fragmentation spectra MS/MS and connected based on spectral similarity. Color map indicates the culture media used in the study as shown in the UpSet plot. Pie charts in the molecular family represent the proportion of fragmentation spectra MS/MS per media. Chemical structures of members of the molecular families corresponding to actinomycins, antimycins and josamycins are shown.


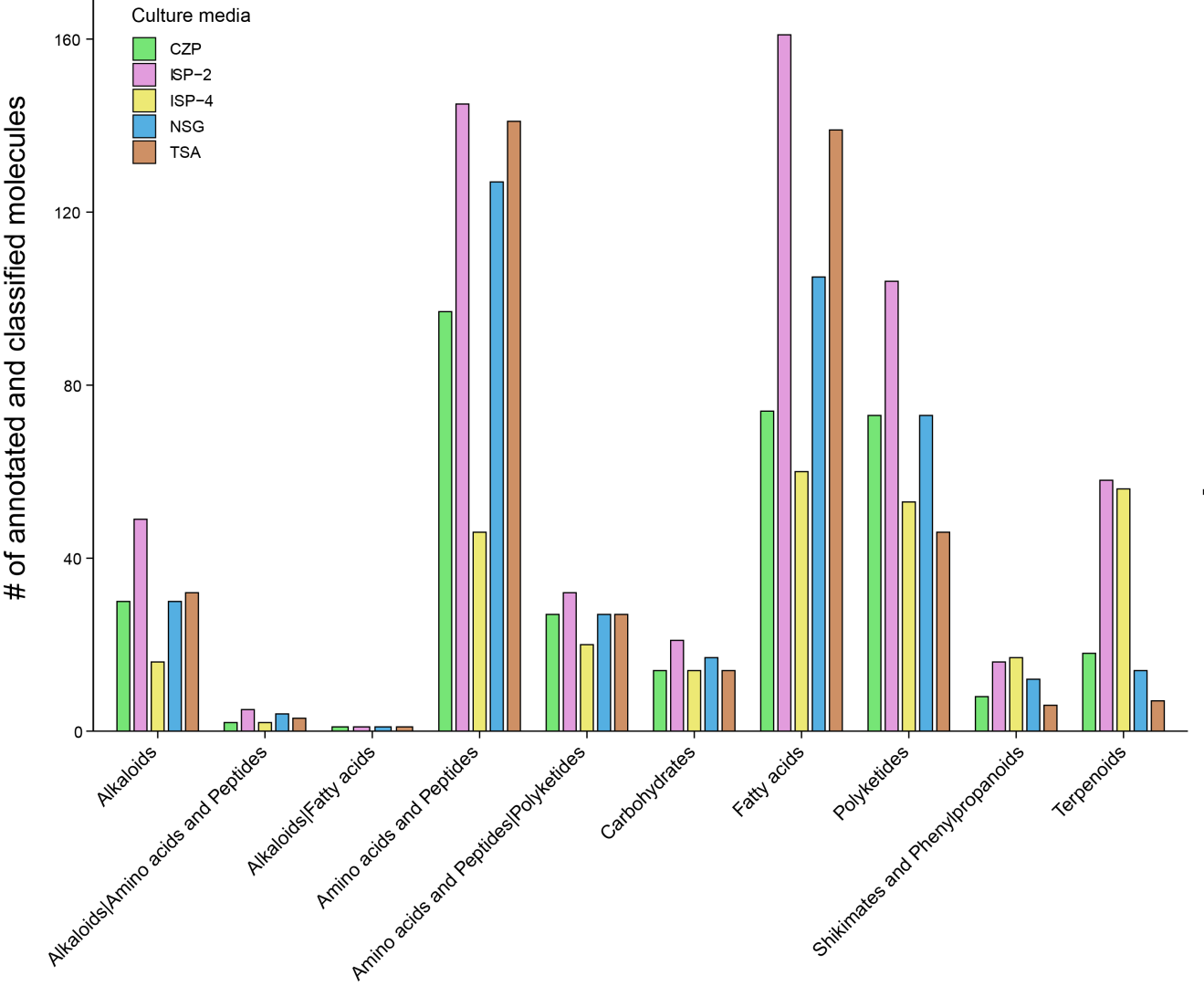


**Supplementary Figure S2.** Number of annotated spectra using GNPS spectral libraries and classified by biosynthetic pathways (NPclassifier) detected in the microbial datasets included in this study. Bar plots showing the variation of the annotated molecules by five of the culture media. Color code as shown in the figure per culture condition.


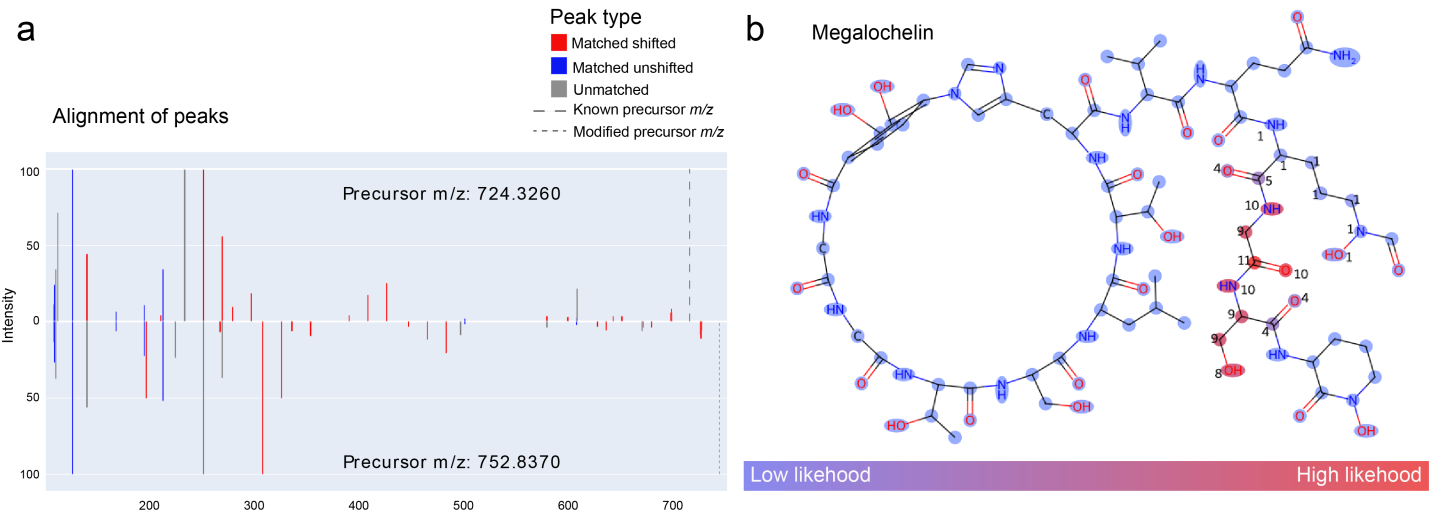


**Supplementary Figure S3**. Prediction of modification site of recently discovered siderophores from *Streptomyces* sp. **a.** At the left, mirror comparison of MS/MS spectra of megalochelin (top) and edaphochelin A (Behsaz *et al.*, submitted) (bottom). Peak type refers to whether a fragment matches exactly (Matched unshifted, in blue) or if shifted by the glycine residue value (Matched shifted, in red); **b.** Chemical structure of megalochelin indicates the probability that the modification (additional glycine residue) is located at a specific site (see NH labeled as 10, in red indicating high likelihood for the modification site to be located).
